# TLRs, NF-*κ*B, JNK, and Liver Regeneration

**DOI:** 10.1155/2010/598109

**Published:** 2010-09-26

**Authors:** Yuji Iimuro, Jiro Fujimoto

**Affiliations:** Department of Surgery, Hyogo College of Medicine, 1-1 Mukogawa-cho, Nishinomiya 663-8501, Japan

## Abstract

While hepatocytes rarely undergo proliferation in normal livers, they quickly induce proliferation in response to loss of liver mass by toxin or inflammation-induced hepatocyte injury, trauma, or surgical resection, leading to a restoration of liver mass to its original size. Recent studies suggest that Toll-like receptor (TLR) signaling participates in this regenerative response. Myeloid differentiation factor (MyD88), a common adaptor molecule in the TLR, IL-1 and IL-18 receptor signaling, plays a key role, at least, in the early phase of liver regeneration. Currently, definite ligands which bind to TLRs and initiate this process are still unclear. TLRs stimulated by their corresponding ligands, as well as tumor necrosis factor (TNF) receptors (TNFRs), can activate downstream signal molecules, including transcription factor nuclear factor (NF)-*κ*B and c-Jun N-terminal kinase (JNK). Previous studies have revealed the important role of TNF receptor signaling, NF-*κ*B, and JNK in liver regeneration by using hepatocyte-specific gene-modified animals. This review will summarize the current knowledge of TLR signaling and their related molecules in liver regeneration. We will also discuss whether modulating these factors may become new therapeutic strategies to promote liver regeneration in various clinical situations.

## 1. Introduction

The liver is a unique organ that has a great ability to regenerate by itself [1, 2]. After loss of considerable liver mass, orchestrated biological responses are quickly activated to restore the loss of liver mass by hepatocyte regeneration until the liver reaches its original size [1–4]. In this regenerative process, mainly mature hepatocytes proliferate by the stimulation of the regenerative factors released from parenchymal and nonparenchymal liver cells. However, liver failure and massive hepatocyte death (apoptosis and necrosis) in fluminant hepatitis could stimulate liver stem cells (or oval cells), which contribute to liver regeneration [5]. Suppressed proliferative ability in mature hepatocytes caused by hepato-toxin exposures, hepatitis B or C virus infection, or excessive lipid accumulation in alcoholic steatohepatitis (ASH), and nonalcoholic steatohepatitis (NASH), also induces the activation of liver stem cells leading to liver regeneration [5]. Because liver injuries induced by inflammation or ischemia/reperfusion and their relation to toll-like receptors (TLRs) are being discussed elsewhere in this issue, we will focus on the role of TLR signaling in liver regeneration after partial hepatectomy (PH), which is a pure hepatic regeneration model. We will further discuss the potential role of TLRs and subsequent signaling pathways that activate nuclear factor (NF)-*κ*B and c-Jun N-terminal kinase (JNK) in liver regeneration.

## 2. Immediate-Early Gene Expression and Signal Transduction after Partial Hepatectomy

 Among a number of biological responses after PH, a group of genes, so called immediate-early genes, are quickly induced [6, 7], which lead to the transition of cell cycle from G0 to G1 in hepatocytes. In contrast, some group of genes are upregulated during the entire period of regenerative responses [8, 9]. Meanwhile, importance of several proinflammatory cytokines including tumor necrosis factor (TNF)-*α* and interleukin (IL)-6 in liver regeneration has been debated during the last decade [2–4]. It is now accepted that these cytokines play significant roles in the priming and early stage of liver regeneration after PH. Although expression of TNF-*α* and IL-6 is upregulated 1 hr after PH, activation of transcription factor NF-*κ*B and AP-1 and expression of immediate-early genes, including c-jun, c-fos, and c-myc, are upregulated within 30 min after PH [7]. These findings suggest that PH induces very quick biological responses, which lead to activation of NF-*κ*B and AP-1, and expression of immediate-early genes. On the other hand, quick upregulation of urokinase-type plasminogen activator (u-PA) and uPA receptor (uPAR) is induced in response to PH [10], implying significant roles in the interaction between u-PA and uPAR in the priming phase of liver regeneration. u-PA also activates several growth factors, including hepatocyte growth factor (HGF) and ligands for epithelial growth factor receptor (EGFR), which induce strong regenerative responses in the liver [11]. 

Many investigators have proposed that gut microflora-derived lipopolysaccharide (LPS) might trigger the initiation of liver regeneration [12–14]. Upon PH, gut-derived LPS might activate nonparenchymal liver cells, particularly Kupffer cells, in which NF-*κ*B is activated. Then, activated Kupffer cells release regenerative cytokines, such as TNF-*α* and IL-6. IL-6 activates STAT3, resulting in the initiation of liver regeneration after PH [2]. After the discovery of TLRs, gut-derived lipopolysaccharide (LPS), a ligand for TLR4, was reconsidered as a trigger of the initiation of liver regeneration [15, 16].

## 3. Toll-Like Receptors and Liver Regeneration

 Toll-like receptors (TLRs) were originally identified as homologs of *Drosophila* Toll that regulates dorsoventral embryonic polarity and antifungal immunity [17]. TLRs facilitate innate immune responses for the host defense against microorganisms by recognizing pathogen-associated molecular patterns (PAMPs) [18]. In addition, endogenous components derived from dying host cells, termed damage-associated molecular patterns (DAMPs), can bind and activate TLRs [19, 20]. After the stimulation with corresponding ligands, TLRs relay signals via myeloid differentiation factor (MyD) 88, a common signal adaptor molecule shared by the receptor for IL-1 and IL-18, and all members of TLRs except for TLR3 [18]. This signal transduction leads to activation of NF-*κ*B and results in the production of various proinflammatory cytokines, including TNF-*α* and IL-6. The MyD88-dependent pathway also activates p38 and c-Jun N-terminal kinase (JNK) as well. The TLR/MyD88-mediated biological events are reminiscent of those observations in liver regeneration after PH. We and others have investigated the role of TLR/MyD88-mediated signal transduction in liver regeneration after PH [15, 16]. Both studies demonstrated that in MyD88-deficient mice, induction of immediate-early genes, including c-fos, c-jun, JunB, and c-myc, was greatly diminished [15], and NF-*κ*B activation in Kupffer cells was also subnormal after PH. Production of IL-6 and TNF-*α* and phosphorylation of STAT-3 were completely suppressed in MyD88-deficient mice after PH [15, 16]. However, these studies showed contradictory roles of MyD88 in the regenerative response of hepatocytes after PH. DNA synthesis in hepatocytes significantly delayed in the early phase of liver regeneration in our investigation [15] implies the significant role of MyD88-dependent signaling in the early and priming phase of liver regeneration. Meanwhile, the MyD88-deficient mice showed no delay in hepatocyte replication in the other report [16]. To discuss this discrepancy, we might consider the breeding condition of animals in different institutions. Recent reports demonstrated that mice from different animal suppliers have different composition of microflora in the intestine [21]. In addition, MyD88-deficient mice are more sensitive to changes in the composition of microflora [22]. Here, we propose that there is a large contribution of intestinal microflora-derived components, as the ligands for TLRs, to liver regeneration after PH. The differences in the composition of gut microflora might lead to the different regenerative responses between different institutions. Nowadays, the concept that TNF*α*, IL-6, and NF-*κ*B are crucial for PH-mediated liver regeneration is still supported by many investigators. Thus, we confidently propose the concept that the MyD88-dependent regenerative response after PH is important for initiating the priming of liver regeneration. Nevertheless, liver mass was finally restored in MyD88-deficient mice compared to WT mice even in our experiment, suggesting that MyD88-independent compensatory regenerative processes also contribute to liver regeneration. 

Another interesting finding is that the intact liver regeneration is induced in *TLR2^−/−^, TLR4^−/−^* [16],* TLR2^−/−^ TLR4^−/−^*, and *TLR9^−/−^* mice [15] after PH. This result suggests that the gut-derived LPS and its receptor TLR4 do not play a major role in triggering the priming phase of liver regeneration. In addition, both IL-1 and IL-18 receptor signalings also use MyD88 as a signal adaptor molecule [23]. Therefore, possible contribution of IL-1 and IL-18 to the priming of liver regeneration should be considered. However, *Caspase-1^−/−^* mice, which lack the capacity to convert proform of IL-1*β* and IL-18 to active form [24, 25], exhibit normal liver regeneration after PH, suggesting minor roles of IL-1*β* and IL-18 in liver regeneration [15]. What is the crucial trigger of the MyD88-dependent biological events in the priming of liver regeneration? We propose that various gut microflora-derived components and/or unidentified endogenous ligands for TLRs activate multiple TLRs including TLR2, 4, 5, and 9 that lead to triggering the priming of liver regeneration after PH. Further investigation is required for proving this hypothesis.

The studies by others and us suggest that TLR/MyD88 signaling participates in the process of liver regeneration, especially in the early and priming phase, after PH. However, definite TLR ligands responsible for the priming process are still unknown. Further investigations are required for addressing this issue. Notably, LPS injection suppresses liver regeneration after PH, indicating that excessive TLR signaling inhibits this regenerative process [26]. Thus, the TLR signaling has double-edge sword-like functions in liver regeneration, and an appropriate magnitude of TLR signaling is required for intact liver regeneration.

 On the other hand, TLR3 utilizes another adaptor molecule, TRIF (TIR domain-containing adaptor-inducing interferon-*β*). Recently, the role of TLR3 signaling in liver regeneration has been reported [27]. In *TLR3^−/−^* mice, initiation of liver regeneration is shifted to the earlier time point, suggesting that TLR3 signaling inhibits the initiation of liver regeneration. This inhibitory effect of TLR3 signaling is also supported by a previous report that activation of TLR3 by injection of its ligand, polyinosinic-polycytidylic acid (poly (I : C)), suppressed liver regeneration after PH [28]. Interestingly, NF-*κ*B activation in hepatocytes was significantly suppressed up to 10 hrs, but its activation was relatively prominent in Kupffer cells in *TLR3^−/−^* mice after PH. These findings suggest that after PH, unknown ligands, probably endogenous ligands for TLR3, activate NF-*κ*B through Rip1 in hepatocytes that inhibit hepatocyte replication. 

 Other components of the innate immune system, complements including C3 and C5, have been reported to participate in liver regeneration after PH [29]. C3- or C5-deficient mice exhibited high mortality, liver parenchymal cell damage, and impaired liver regeneration after PH. NF-*κ*B activation was markedly reduced at 1 hr after PH in C3-deficient mice. Thus, the complement system plays a significant role in the priming phase as well as in the subsequent proliferative phase during liver regeneration. 

## 4. Roles of NF-*κ*B in Liver Regeneration after Partial Hepatectomy

 NF-*κ*B is an essential transcription factor for maintaining liver homeostasis, including cell survival and death [30]. As NF-*κ*B p65 whole body knockout mice cause the embryonic death due to an extensive hepatocyte apoptosis, NF-*κ*B plays a crucial role in preventing hepatocyte apoptosis during liver development [31]. 

A very quick increase in NF-*κ*B DNA binding activity after PH has been reported in early 1990s [32, 33]. We are the first demonstrating the role of NF-*κ*B in liver regeneration after PH. We introduced I*κ*B *α* superrepressor, a potent NF-*κ*B inhibitor, by adenoviral vectors (Ad-I*κ*Bsr) to inhibit NF-*κ*B activation in the liver [34]. In this study, the rat livers introducing superrepressor of I*κ*B *α* (I*κ*Bsr) demonstrated the prominent hepatocyte apoptosis and blunted early increase in NF-*κ*B DNA binding activity in hepatocytes during the initial 24 hrs after PH, suggesting antiapoptotic role of NF-*κ*B in hepatocytes after PH. Liver regeneration was significantly impaired in this study. A similar experiment employing Ad-I*κ*Bsr in mice was reported by Yang et al. [35]. In this paper, overexpression of I*κ*Bsr in mouse livers also inhibited liver regeneration after PH but did not induce hepatocyte apoptosis. Discrepancy between these reports might be due to different infectious abilities against adenoviral vectors between rats and mice. This might induce hepatocyte apoptosis only in rats. Since TNF*α* is produced in the early period after PH, this discrepancy also might be due to different sensitivity to TNF*α*-induced cell death between rats and mice. Thus, activation of NF-*κ*B in hepatocytes is important as an antiapoptotic factor, at least in rats, but not in mice after PH. 

A subsequent study further reported the specific role of hepatocyte NF-*κ*B in liver regeneration after PH [36]. Transgenic mice expressing a hepatocyte-specific mutant I*κ*B*α* exhibited the normal regenerative response and did not show hepatocyte apoptosis after PH. TNF*α* treatment induced prominent apoptosis in these animals. A use of adenoviral vector in our study, indeed, possibly made the issue complicated because adenoviral vectors could infect not only hepatocytes, but also nonparenchymal liver cells including Kupffer cells. In the study by Maeda et al. [37] to inhibit NF-*κ*B activation, IKK*β* (inhibitor of kappaB kinase *β*, also known as IKK2) was inactivated in both hepatocytes and hematopoietic-derived cells (Kupffer cells) by conditional knockout technique using Mx-1 Cre transgenic mice. These mice reduced the proliferative response after PH. Taken together, these findings suggest that NF-*κ*B activation in Kupffer cells is more important than that in hepatocytes. 

In a recent study [38], the modulation of hepatocyte NF-*κ*B activity by inactivation of IKK*β* demonstrated the controversial results from the studies by Maeda et al., [37], Yang et al., [35], and us [34]. Hepatocyte-specific IKK*β* knockout mice showed that accelerated hepatocyte proliferation and early NF-*κ*B activation in nonparenchymal liver cells including Kupffer cells were observed. In contrast, a weak and delayed NF-*κ*B activation in hepatocytes was seen after PH, suggesting that this IKK and NF-*κ*B activation in hepatocytes might be through IKK*α*. These findings further suggest that IKK*α* in hepatocytes is crucial in liver regeneration after PH. Addressing this issue, further investigation is needed. JNK activation in hepatocyte-specific IKK*β* knockout mice is prolonged in the liver after PH [38]. Because Cyclin D is a JNK/AP-1 target gene, a strong and sustained JNK activation could be involved in the accelerated liver regeneration by inducing Cyclin D expression in hepatocyte-specific IKK*β* knockout mice. This study also examined pharmacological inhibition of IKK*β*, which enables to inhibit IKK*β* activity in both hepatocytes and nonparenchymal cells. This treatment had little effect on the regenerative process [38], which corroborates the results from Chaisson et al. [36], but it does not support the results from Maeda et al., Yang et al., and us; NF-*κ*B in Kupffer cells is critical in liver regeneration. 

The crosstalk between NF-*κ*B and JNK in the liver after PH has been demonstrated in the study using Gadd45*β* (growth arrest and DNA-damage-inducible gene 45*β*), an NF-*κ*B target gene, knockout mice [39]. *G* *a* *d* *d*45*β* ^−/−^ mice exhibited decreased hepatocyte proliferation and increased programmed cell death during liver regeneration, in which JNK activity was increased and sustained. This study further supports the concept that an appropriate activation of NF-*κ*B in hepatocytes is important for liver regeneration by regulating JNK activity through Gadd45*β*. This issue will be discussed below. 

 It is now almost acceptable that the activation of NF-*κ*B in Kupffer cells is crucial for intact liver regeneration after PH, especially for the priming phase [3]. Activated NF-*κ*B conducts sequential production of TNF*α* and IL-6, each of which plays a significant role in the priming phase of the regenerative process. Indeed, early activation of NF-*κ*B in liver regeneration after PH was demonstrated to primarily occur in Kupffer cells using *cis*-NF-*κ*B-EGFP transgenic mice [35]. Moreover, inactivation of NF-*κ*B in Kupffer cells as well as in hepatocytes impaired the proliferative response after PH as mentioned above [37].

## 5. JNK Activation after Partial Hepatectomy

 Prompt expression of *c-jun* and activation of c-Jun N-terminal kinase (JNK) have been reported after PH as described above [40, 41]. Fetuses lacking *c-jun *die at midgestation with defects in heart morphogenesis and increased apoptosis of both hepatoblasts and hematopoietic cells in the fetal liver [42, 43]. Moreover, mice lacking the JNK kinase SEK1 exhibit a liver defect similar to *c-jun^−/−^* fetus [44, 45]. Roles of the transcription factor AP-1 (activator protein-1), including c-Jun, as a regulator of cell survival and death are well summarized in a previous paper [46]. The function of c-Jun at later stages of liver development or in adult liver remained to be elucidated. 

The role of c-jun during liver regeneration after PH was first reported by using floxed *c-jun *alle (*c-jun^f^*) mice crossing with Alfp-cre or Mx-cre transgenic mice [47]. In either Alfp-cre or Mx-cre-induced conditional c-jun knockout mice, prominent hepatocyte death and lipid accumulation in hepatocytes were observed after PH. These mice exhibited high mortality and impaired liver regeneration. These results indicate that *c-jun* is required for hepatocytes regeneration after PH. Interestingly, c-Jun N-terminal phosphorylation is not required for c-Jun function in liver regenerative response [47]. 

 Important roles of JNK activation after PH have been reported [48]. Inhibition of JNK activity in the liver using a small molecule JNK inhibitor (SP600125) resulted in reduced c-Jun phosphorylation and AP-1 DNA binding activity. JNK inhibition suppressed cyclin D1 expression and delayed hepatocyte proliferation after PH, resulting in decreased survival but not hepatocyte apoptosis. This implied that JNK drives the transition of cell cycle from G0 to G1 transition in hepatocytes and that cyclin D1 is a crucial target gene of the JNK pathway in liver regeneration. However, these results were different from the data obtained from the study in which c-jun was genetically inactivated [47]. Potential explanation for this discrepancy is that JNK mediates its effects on liver regeneration after PH through other targets such as ATF2 or JunD [48]. As mentioned above, c-Jun N-terminal phosphorylation is not required for regeneration after PH [47], suggesting that increased JNK activity after PH has other significant roles rather than its ability to phosphorylate c-Jun N-terminal. Two isoforms of JNK are expressed in the liver; JNK1 and JNK2 [49, 50]. Distinct functions in these JNKs have been reported [51]. Namely, JNK1 induces cell proliferation with c-Jun phosphorylation. In contrast, JNK2 suppresses proliferation by degradation of c-Jun. Sabapathy and Wagner also reported an acceleration of liver regeneration in *JNK2^−/−^* mice in their perspective [52]. 

 HGF and EGFR-ligands activate JNK. Mice expressing an inducible *Met* mutation by Mx-cre system in the liver showed blunted c-jun phosphorylation around 48 h after PH and a great suppression of Erk1/2 phosphorylation during the entire regeneration process [53]. Hepatocyte-specific EGFR deletion resulted in reduced cyclin D1 expression and delayed hepatocyte proliferation after PH. Surprisingly, this observation was accompanied by sustained activation of c-Jun and reduced NF-*κ*B DNA binding activity [54]. This prolonged c-Jun activation after PH in the hepatocyte-specific EGFR-deleted mice might be independent of EGFR-mediated JNK activation, or there might be unknown negative regulatory mechanism on JNK/c-Jun activation in the EGFR signaling cascade. Nevertheless, it is suggested that prolonged activation of c-Jun inhibits liver regeneration after PH. Prolonged activation of JNK after PH has also been observed in *G* *a* *d* *d*45*β* ^−/−^ mice as mentioned above [39]. Why does prolonged JNK activation suppress liver regeneration after PH? Notably, a genetical ablation of JNK2 prevents impaired liver regeneration and increased hepatocyte cell death in *G* *a* *d* *d*45*β* ^−/−^ mice after PH. Although it is obvious that JNK pathway directly induces cyclin D1 upregulation to contribute to hepatocyte proliferation [39], however, prolonged JNK activation hampers liver regeneration after PH, at least, by enhancing programmed cell death. 

TGF-*β*-activated kinase (TAK)-1 is a crucial component for activating both NF-*κ*B and JNK. An adenoviral overexpression of dominant negative in the liver accelerated the proliferative response after PH [55]. This might be explained by the inhibitory role of IKK*β* and JNK2 in liver regeneration in previous studies [38, 52]. An appropriate crosstalk between JNK and NF-*κ*B is critical for intact liver regeneration after PH. An opposing role of NF-*κ*B and JNK in hepatocarcinogenesis has been proposed. Loss of NF-*κ*B activity in hepatocytes increases the sensitivity to hepatocarcinogenesis with increased JNK activation [37, 56–58] while JNK1 knockout mice exhibit decreased carcinogenesis after N-nitrosodiethylamine administration [59]. The mutual crosstalk between the NF-*κ*B and JNK has been well documented in the previous reviews [60, 61].

**Figure 1:**
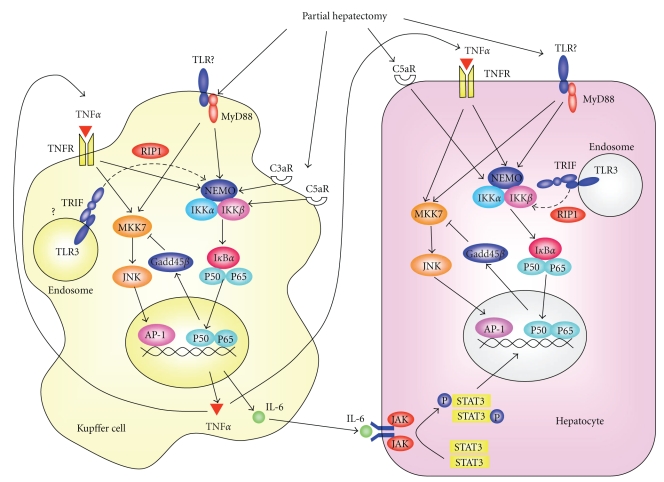
Signal transduction pathways during the priming phase of liver regeneration. Interactions between Kupffer cells and hepatocytes are also illustrated while other nonparenchymal cells are also possibly involved. AP-1, activator protein; C3aR, activated compliment 3 receptor; C5aR, activated compliment 5 receptor; Gadd45*β*, growth arrest and DNA-damage-inducible gene 45*β* *;* IKK, inhibitor of nuclear factor *κ*B kinase; IL-6, interleukin-6; JAK, Janus-associated kinase; JNK, c-jun N-terminal kinase; MKK7, mitogen-activated protein kinase 7; MyD88, myeloid differentiation factor 88; NEMO, nuclear factor *κ*B essential modulator; RIP, receptor interacting protein; TLR, toll-like receptor; TNF*α*, tumor necrosis factor *α*; TRIF, TIR-domain containing adaptor inducing interferon-*β*.

 In summary, the transient upregulation of the two downstream targets NF-*κ*B and JNK in TLR/MyD88 and TNFR signaling is strictly organized in Kupffer cells and hepatocytes upon PH, which leads to normal liver regeneration (Figure 1).

## 6. Clinical Issues in Liver Regeneration

 Understanding the mechanism of liver regeneration is important for managing several clinical conditions, such as acute liver failure and impaired hepatic functions after liver transplantation. An issue concerning small-for-size grafts in liver transplantation is a well-known clinical subject. An experiment using small-for-size graft in rats revealed that suppressed AP-1 DNA binding activity and reduced cyclin D1 expression resulted in impaired liver regeneration. This was attenuated by administration of a radical scavenger [62]. Impaired liver regeneration in patients with NASH (nonalcoholic steatohepatitis) or NAFLD (nonalcoholic fatty liver disease) is also a major clinical issue. Animal experiments revealed that impaired liver regeneration with suppressed NF-*κ*B DNA binding activity and delayed and prolonged *c-jun* expression after PH in ob/ob mice [63]. Microarray analysis in the patient livers with NASH revealed that mRNA expression of transcription factors such as v-Jun (oncogenic isoform of c-Jun) was significantly suppressed compared with nonobese control patients even without liver resection [64]. However, the most critical issue in clinical situation is impaired liver regeneration in patients with advanced liver fibrosis. A number of factors including deposition of excessive amount of extracellular matrix, existence of continuous inflammation, transformation of sinusoidal endothelial cells and hepatic stellate cells, and decreased portal blood flow may affect the regenerative ability in the fibrotic livers. In addition, increased JNK activity, as observed in animal models during fibrogenesis [65], may also account for the impaired regenerative process in these patients. 

## 7. Summary

 The ligands for TLRs are a strong candidate for triggering the initiation of liver regeneration. However, discovery of the real trigger which initiates liver regeneration is a challenging assignment for hepatologists because multiple ligands and multiple TLRs could contribute to this process. In addition, understanding of the interaction between the two major transcription factors; NF-*κ*B and AP-1 (such as c-Jun), and the regulation of JNK activity by NF-*κ*B seems to be critical to elucidate the well-orchestrated process. Further investigation of distinct roles between these factors in hepatocytes and nonparenchymal cells should be required employing cell-specific gene manipulation techniques. The management of the impaired balance between these factors in nonparenchymal cells and hepatocytes may provide insight into developing new strategies for inappropriate hepatic regenerative response in patients with chronic and acute liver diseases.
